# Building Capacity on Hypertension Management in Nigeria

**DOI:** 10.1001/jamanetworkopen.2026.1674

**Published:** 2026-03-06

**Authors:** Shivani Mishra, Anyiekere Ekanem, Daniel Henry, Esther Idang, Ifiok Ituen, Saviour Okon, Dorcas Ekpoudom, Weixi Chen, Deborah Onakomaiya, Nafesa Kanneh, Daphne Lew, Erinn M. Hade, Angela A. Aifah, Eno Angela Attah, Gbenga Ogedegbe, Dike Ojji

**Affiliations:** 1 Institute for Excellence in Health Equity, New York University Grossman School of Medicine, New York; 2 Department of Community Medicine, University of Uyo, Uyo, Akwa Ibom State, Nigeria; 3 Cardiovascular Research Unit, University of Abuja Teaching Hospital, Abuja, Nigeria; 4 Department of Population Health, New York University Grossman School of Medicine, New York; 5 Center for Biostatistics and Data Science, Washington University School of Medicine, St Louis, Missouri; 6 Akwa Ibom State Primary Healthcare Development Agency, Uyo, Akwa Ibom, Nigeria; 7 Department of Internal Medicine, Faculty of Clinical Sciences, University of Abuja, Abuja, Nigeria

## Abstract

This cross-sectional study estimates changes in health care workers’ knowledge of the simplified Nigerian hypertension treatment protocol after participating in a hypertension management training program.

## Introduction

Strengthening primary care for noncommunicable disease management through task-sharing is critical to reducing premature mortality in low- and middle-income countries.^1^ Although task-shifting guidelines for hypertension exist in Nigeria,^2^ primary health care workforces remain underprepared, reflecting their long-standing focus on infectious diseases.^3^

We implemented the Hypertension Academy training program in Akwa Ibom State, Nigeria, as part of the MAP-IT study to support uptake of the simplified Nigerian hypertension treatment protocol^2^ among health workers. The primary objective was to estimate short-term changes in cadre-specific hypertension knowledge before and after training. Secondary objectives were to assess training acceptability and self-reported confidence in hypertension management.

## Methods

 This cross-sectional study evaluated the outcomes of 3 Hypertension Academy workshops conducted between January 16 and April 20, 2024, for nurses, community health extension workers (CHEWs), primary care physicians, and community pharmacists. The study was approved by the institutional review boards of the University of Abuja and New York University Grossman School of Medicine. All participants provided written informed consent, and analytic datasets contained no individually identifiable information. The study followed the STROBE reporting guideline for cross-sectional studies.

The Hypertension Academy targeted proximal implementation outcomes through structured, guideline-based training, focusing on short-term knowledge gains and acceptability rather than downstream clinical outcomes. Training content was based on the simplified Nigerian hypertension protocol for primary care^2^ and the World Health Organization HEARTS package.^4^ Participants completed an anonymized pretraining and posttraining knowledge assessment (eAppendix in Supplement 1) and a posttraining evaluation of content, delivery, and applicability. The knowledge assessment included 10 items covering core domains of primary care hypertension management. Pretraining and posttraining assessments were linked using unique codes for matching, and analyses were conducted on deidentified paired data. Scores ranged from 0 to 10, with higher scores indicating greater guideline-based knowledge. Training evaluations assessed content relevance, delivery, and applicability using 5-point Likert scales from 1 (poor) to 5 (excellent) and were summarized descriptively using means, SDs, and response distributions, with domain-level summaries characterizing acceptability.

Normality of pretraining and posttraining differences was assessed using the Shapiro-Wilk test.^5^ Because differences were nonnormally distributed, results were summarized using medians and IQRs. Paired comparisons were by the Wilcoxon signed rank test,^6^ with effect sizes reported as Hodges-Lehmann median differences and CIs. Bonferroni adjustment maintained a 95% family-wise confidence level. Analyses were stratified by cadre to estimate within-cadre change; multilevel models and workshop-level clustering adjustments were not applied. The analyses were conducted using R, version 4.5.1 (R Foundation).

## Results

A total of 236 health workers (169 female [71.6%] and 67 male [28.4%]) participated in the training and were included in the primary analysis, including 102 nurses and CHEWs (43.2%), 95 pharmacists (40.3%), and 39 physicians (16.5%). Within-participant improvements were found in hypertension knowledge across cadres, with higher median posttest scores in each group (from 7.0 [IQR, 6.0-8.0] for nurses and CHEWS to 9.0 [IQR, 8.0-9.0) for physicians) (Table 1).

**Table 1.** Comparison of Knowledge of Hypertension Among the Different Categories of Health Workers (N = 236)

Workforce cadre	Participants, No. (%)	Knowledge scores, median (IQR)		Difference (95% CI)
		Pretest	Posttest	
Nurses and CHEWs	102 (43.2)	6.5 (5.5-7.5)	7.0 (6.0-8.0)	0.8 (0.3-1.2)
Pharmacists	95 (40.3)	4.0 (3.0-5.5)	7.5 (6.3-8.0)	3.5 (3.0-4.0)
Physicians	39 (16.5)	6.5 (5.0-8.0)	9.0 (8.0-9.0)	2.2 (1.5-3.0)

Abbreviation: CHEW, community health extension worker.

Training evaluations showed high acceptability, with median Likert scores for content relevance, delivery, and applicability ranging from 4.0 to 5.0. Participants also reported increased confidence in hypertension management and high ratings of applicability of the training to their clinical roles (Table 2).

**Table 2.** Evaluation of Hypertension Workshop by Health Workers

Survey domain^a^	Median (IQR)	Participants, No. (%)				
		Excellent	Very good	Neutral	Good	Poor
**Nurses and CHEWs**						
Content relevance	4.0 (4.0-5.0)	32 (36.0)	40 (44.9)	1 (1.1)	16 (18.0)	0
Delivery (methodology)	4.0 (4.0-5.0)	31 (38.8)	33 (41.3)	2 (2.5)	14 (17.5)	0
Delivery (role play)	4.0 (4.0-5.0)	36 (40.0)	37 (41.1)	2 (2.2)	15 (16.7)	0
Applicability	5.0 (4.0-5.0)	26 (52.0)	18 (36.0)	1 (2.0)	5 (10.0)	0
**Pharmacists**						
Content relevance	5.0 (5.0-5.0)	85 (78.7)	23 (21.3)	1 (0.9)	0	0
Delivery (methodology)	5.0 (5.0-5.0)	90 (84.9)	16 (15.1)	0	0	0
Delivery (role play)	5.0 (4.0-5.0)	50 (56.8)	32 (36.4)	5 (5.7)	1 (0.17)	0
Applicability	5.0 (5.0-5.0)	87 (84.5)	16 (15.5)	0	0	0
**Physicians**						
Content relevance	5.0 (4.0-5.0)	19 (63.3)	10 (33.3)	0	0	1 (3.3)
Delivery (methodology)	5.0 (4.0-5.0)	21 (70.0)	8 (26.7)	0	0	1 (3.3)
Delivery (role play)	5.0 (4.0-5.0)	18 (60.0)	11 (36.7)	0	0	1 (3.3)
Applicability	5.0 (4.3-5.0)	22 (73.3)	7 (23.3)	0	0	1 (3.3)

Abbreviation: CHEW, community health extension worker.

^a^ Full survey items are provided in the eAppendix in Supplement 1.

## Discussion

This cross-sectional study found short-term improvements in hypertension knowledge and high training acceptability among primary care health workers participating in the Hypertension Academy. Use of simplified, context-specific protocols^2,4^ and interactive training aligns with global recommendations for task-sharing in low- and middle-income countries.

Several limitations merit consideration. The pretraining and posttraining design without a control group limited causal inference. Identical assessments may have introduced test familiarity effects, and posttraining evaluations may have reflected social desirability bias. Response rates varied across cadres, and the single-state implementation limits generalizability. Despite these limitations, the Hypertension Academy represents a scalable approach to primary care workforce development. Future studies should assess knowledge retention, clinical outcomes, and integration into routine continuing education systems.

## Notes

Supplement 1. **eAppendix.** Cadre-Specific Pretraining and Posttraining Surveys

Supplement 2.
Data Sharing Statement
