# Supplementary material for: Building Capacity on Hypertension Management in Nigeria
Source: JAMA Netw Open. 2026 Mar 6;9(3):e261674. doi: 10.1001/jamanetworkopen.2026.1674 (PMC12966916; doi:10.1001/jamanetworkopen.2026.1674)
Supplement: Supplement 1. — eAppendix. Cadre-Specific Pretraining and Posttraining Surveys [file jamanetwopen-e261674-s001.pdf]

Supplemental Online Content

Mishra S, Ekanem A, Henry D, et al. Building capacity on hypertension management in Nigeria. *JAMA Netw Open*. 2026;9(3):e261674. doi:10.1001/jamanetworkopen.2026.1674

eAppendix. Cadre-Specific Pretraining and Posttraining Surveys

This supplemental material has been provided by the authors to give readers additional information about their work.

© 2026 Mishra S et al. *JAMA Network Open*.

eAppendix. Cadre-Specific Pretraining and Posttraining Surveys

Managing Hypertension Among People Living With HIV: An Integrated Model (Map-It): Nurses & Community Health Extention Workers' Pre/Post Test

☐ Participant ID:_________________________________

Attempt all

1). High Blood Pressure and Hypertension is same thing_?

a) True b) False c) Not Sure

2). Blood Pressure values can be said to be **elevated or high** when the BP value (excludes co-morbidities) is:

a). More than or equal to140 mmHg and 90mmHg

b). More than or equal to 130mmHg and 80mmHg

c). More than or equal to 120 mmHg and 70mmHg

3). When a BP reading is elevated what is the next line of action?

a). Provide counsel to the person and send them home

b). Refer person to community nurses in the health center

c). Provide counselling and refer the person to community nurses in the health center

4). When discussing the importance of hypertension treatment, the followingfacts can be used to support your case:

a) Hypertension treatment can be practical and uncomplicated when aprecise protocol is used.

b) Hypertension treatment is needed because currently there is no way to predict which patients will have a heart attack or stroke.

c) Hypertension treatment is affordable. The medicines to treat hypertension are inexpensive.

d) All of the above

5). Anthropometric measurement includes:

a). Body mass index, blood pressure measurement

b). Blood Pressure measurement, Body temperature

c). Weight, height, Waist circumference, Hip ratio

6). How many times should you measure blood pressure when a patient is seen for a clinic appointment?

a). Once correctly

b) Three times with average of first 2 measurements

c). The number does not matter as long as I get a value

c). Twice correctly

© 2026 Mishra S et al. *JAMA Network Open*.

7). The following steps are related to applying the blood pressure cuff. Which step is **NOT CORRECT**?

a). Place their arm on the table, relaxed with palm facing upward.

b). Make sure the arm cuff is slightly inflated before placing it around the patient's upper arm.

c). Wrap the cuff comfortably or snugly above the elbow, about 2cm (or 2 finger widths) from the elbow and secure the Velcro tape.

d). Keep the cuff at the level of the heart during measurement. The tubing should fall over the front centre of the elbow crease, if it is positioned correctly.

8). In reviewing the practices at your clinic, you recognize that medication adherence and/or clinic visits by patients have been low. List two practices that can be implemented to improve adherence.

i---

ii---

9). List two hypertension management tools or equipment used in a clinic

a). ---

b). --

10). In the treatment of hypertension in Nigerians, which of the following is true?

a). Majority of patients require just one medication

b). Most patients require more than one medication

c). Atenolol is very effective as monotherapy

d). Angiotensin converting enzyme inhibitors (ACEIs) are effective as monotherapy

e). Thiazide diuretics are more efficacious than calcium channel blockers as monotherapy

© 2026 Mishra S et al. *JAMA Network Open*.

Managing Hypertension Among People Living With HIV: An Integrated Model (Map-It): Physicians And Pharmacists' Pre/Post Test

Participant ID:_______________________________________

1). Blood Pressure values can be said to be **elevated or high** when the BP value (excludes co-morbidities) is:

a). More than or equal to 140 mmHg and 90mmHg

b). More than or equal to 130mmHg and 80mmHg

c). More than or equal to 120 mmHg and 70mmHg

d). More than or equal to 135mmHg and 85mmHg

2). How many times should you measure blood pressure when a patient is seen for a clinic appointment?

a). Once correctly

b) Three times with average of last 2 measurements

c). The number does not matter as long as I get a value

d). Twice correctly.

3). The following steps are related to applying the blood pressure cuff. Which step is **NOT CORRECT**?

e) Place their arm on the table, relaxed with palm facing upward.

f) Make sure the arm cuff is slightly inflated before placing it around the patient's upper arm.

g) Wrap the cuff comfortably or snugly above the elbow, about 2cm (or 2 finger widths) from the elbow and secure the Velcro tape.

h) Keep the cuff at the level of the heart during measurement. The tubing should fall over the front centre of the elbow crease if it is positioned correctly.

4). In reviewing the practices at your clinic, you recognize that medication adherence and/or clinic visits by patients have been low. List two practices that can be implemented to improve adherence.

i---

ii--

5). In the treatment of hypertension in Nigerians, which of the following is true?

a) Majority of patients require just one medication

b) Most patients require more than one medication

c) Atenolol is very effective as monotherapy

d) Angiotensin converting enzyme inhibitors (ACEIs) are effective as monotherapy

e) Thiazide diuretics are more efficacious than calcium channel blockers as monotherapy

6). The current prevalence of hypertension in Nigeria stands at about?

a. a.10-20%

b. b. 5-10%

© 2026 Mishra S et al. *JAMA Network Open*.

c. c. 25-40%
d. d. 40-50%
e. e. 45-55%

7). What is mean arterial pressure?

a. The difference between systolic BP and diastolic BP
b. The sum of systolic BP and diastolic BP
c. The sum of pulse pressure + ½ diastolic BP
d. The sum of diastolic BP + ½ pulse pressure
e. The sum of diastolic BP + 1/3 pulse pressure

8). A 55-year-old man with background hypertension on 5mg of Amlodipine was found to have a BP reading of 170/100mmHg. **How should he be managed?**

a. Increase Amlodipine to 10mg
b. Change Amlodipine to Moduretic once daily
c. Change Amlodipine to Lisinopril 20mg once daily
d. Add Tab Telmisartan 80mg to Amlodipine 5mg
e. Commence single pill combination of Amlodipine +Telmisartan 10/80 mg once daily

9). Which of the following is NOT an advantage of using single-pill anti-hypertensive combination therapy

a. Leads to simplicity of treatment
b. Improves compliance
c. Improves tolerability
d. Reduces the price of medication
e. It improves flexibility

10). Which of the following is the most metabolically neutral anti-hypertensive combination therapy?

a. Calcium channel blockers plus Thiazide diuretics
b. Calcium channel blockers plus Moduretic
c. Calcium channel blockers plus RAS blockers
d. Beta blockers plus Thiazide diuretics
e. RAS Blockers plus Thiazide-like diuretic

© 2026 Mishra S et al. *JAMA Network Open*.
