# Supplementary material for: Building Capacity on Hypertension Management in Nigeria
Source: JAMA Netw Open. 2026 Mar 6;9(3):e261674. doi: 10.1001/jamanetworkopen.2026.1674 (PMC12966916; doi:10.1001/jamanetworkopen.2026.1674)
Supplement: Supplement 2. — Data Sharing Statement [file jamanetwopen-e261674-s002.pdf]

Data Sharing Statement

Data
Data available: Yes
Data types: Deidentified participant data
How to access data: De-identified aggregate tables supporting the findings of this study are publicly available in the Harvard Dataverse at https://doi.org/10.7910/DVN/Z4VTYB.
When available: With publication

Supporting Documents
Document types: Other (please specify)
Additional Information: Survey used for the study, and STROBE checklist
How to access documents: These documents are available as supplemental information to the article.
When available: With publication

Additional Information
Who can access the data: Researchers whose proposed use of the data has been approved.
Types of analyses: For any purpose
Mechanisms of data availability: After approval of a proposal and with a signed data access agreement
